# *N*′-(5-Bromo-2-hydroxy­benzyl­idene)-4-chloro­benzohydrazide

**DOI:** 10.1107/S160053681000752X

**Published:** 2010-03-06

**Authors:** Cong-Shan Zhou, Tao Yang

**Affiliations:** a College of Chemistry and Chemical Engineering, Hunan Institute of Science and Technology, Yueyang, Hunan 414006, People’s Republic of China

## Abstract

The title Schiff base, C_14_H_10_BrClN_2_O_2_, exists in a *trans* configuration with respect to the C=N bond and the dihedral angle between the two benzene rings is 0.8 (2)°. There is an intra­molecular O—H⋯N hydrogen bond in the mol­ecule, which generates an *S*(6) loop. In the crystal, inter­molecular N—H⋯O hydrogen bonds link adjacent mol­ecules into extended chains propagating along the *c*-axis direction.

## Related literature

For background to the biological properties of Schiff bases, see: Ritter *et al.* (2009 ▶); Bagihalli *et al.* (2008 ▶). For related structures, see: Fun *et al.* (2008 ▶); Shafiq *et al.* (2009 ▶); Goh *et al.* (2010 ▶). Zhou *et al.* (2009 ▶); Zhou & Yang (2009 ▶, 2010*a* ▶,*b* ▶).
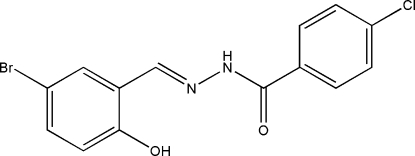


## Experimental

### 

#### Crystal data

C_14_H_10_BrClN_2_O_2_
*M* *_r_* = 353.60
Monoclinic, 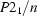

*a* = 5.893 (2) Å
*b* = 31.708 (11) Å
*c* = 7.437 (3) Å
β = 92.017 (8)°
*V* = 1388.8 (9) Å^3^
*Z* = 4
Mo *K*α radiation
μ = 3.15 mm^−1^
*T* = 298 K
0.17 × 0.15 × 0.15 mm

#### Data collection

Bruker SMART 1000 CCD diffractometer
Absorption correction: multi-scan (*SADABS*; Sheldrick, 1996 ▶) *T*_min_ = 0.616, *T*_max_ = 0.649
7670 measured reflections
2661 independent reflections
1555 reflections with *I* > 2σ(*I*)
*R*_int_ = 0.043

#### Refinement

*R*[*F* ^2^ > 2σ(*F* ^2^)] = 0.041
*wR*(*F* ^2^) = 0.119
*S* = 1.02
2661 reflections
185 parameters
1 restraint
H atoms treated by a mixture of independent and constrained refinement
Δρ_max_ = 0.58 e Å^−3^
Δρ_min_ = −0.51 e Å^−3^

### 

Data collection: *SMART* (Bruker, 2007 ▶); cell refinement: *SAINT* (Bruker, 2007 ▶); data reduction: *SAINT*; program(s) used to solve structure: *SHELXTL* (Sheldrick, 2008 ▶); program(s) used to refine structure: *SHELXTL*; molecular graphics: *SHELXTL*; software used to prepare material for publication: *SHELXTL*.

## Supplementary Material

Crystal structure: contains datablocks global, I. DOI: 10.1107/S160053681000752X/hb5343sup1.cif

Structure factors: contains datablocks I. DOI: 10.1107/S160053681000752X/hb5343Isup2.hkl

Additional supplementary materials:  crystallographic information; 3D view; checkCIF report

## Figures and Tables

**Table 1:** Hydrogen-bond geometry (Å, °)

*D*—H⋯*A*	*D*—H	H⋯*A*	*D*⋯*A*	*D*—H⋯*A*
O1—H1⋯N1	0.82	1.93	2.642 (4)	145
N2—H2⋯O2^i^	0.90 (1)	1.96 (2)	2.829 (3)	163 (4)

Symmetry code: (i) 
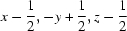
.

**Fig. 1.**
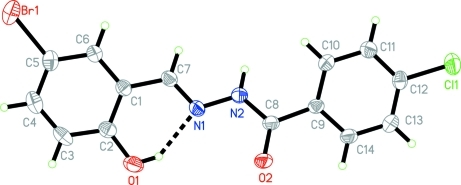
The molecular structure of (I), with ellipsoids drawn at the 30% probability level.

**Fig. 2.**
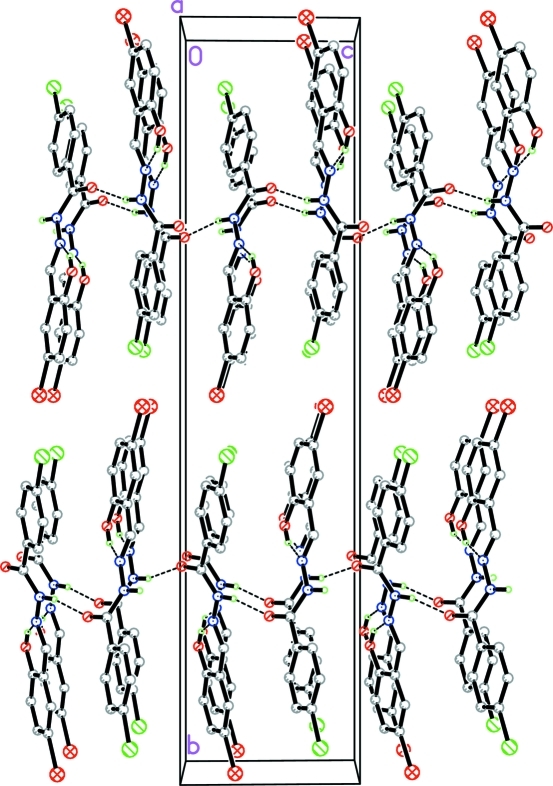
The packing of (I), viewed along the a axis. Hydrogen bonds are drawn as dashed lines.

## supplementary crystallographic information

### Comment

Some Schiff bases possess biological properties, such as antibacterial, antimicrobial, and antitumor activities (Ritter *et al.*, 2009; Bagihalli *et al.*, 2008). Recently, a large number of Schiff bases derived from the reaction of aldehydes with benzohydrazides have been reported (Fun *et al.*, 2008; Shafiq *et al.*, 2009; Goh *et al.*, 2010). As a continuation of these studies, in this paper, the crystal structure of the title Schiff base, (I), derived from the condensing of 5-bromo-2-hydroxybenzaldehyde with 4-chlorobenzohydrazide in methanol is reported.

In the title compound, Fig. 1, all the bond lengths are comparable with those observed in other similar compounds (Zhou *et al.*, 2009; Zhou & Yang, 2009; Zhou & Yang, 2010a,b). The molecule exists in a *trans* configuration with respect to the acyclic C═N bond. There is an intramolecular O—H···N hydrogen bond in the molecule (Table 1). The dihedral angle between the two benzene rings is 0.8 (2)°.

In the crystal structure, intermolecular N—H···O hydrogen bonds link adjacent molecules into extended chains along the *c* axis (Table 1 and Fig. 2).

### Experimental

5-Bromo-2-hydroxybenzaldehyde (1.0 mmol, 201 mg) and 4-chlorobenzohydrazide (1.0 mmol, 170 mg) were dissolved in a methanol solution (30 ml). The mixture was stirred for 30 min at room temperature. The resulting solution was left in air for a few days, yielding colourless blocks of (I).

### Refinement

H2 attached to N2 was located in a difference map and refined with N—H distance restrained to 0.90 (1)Å. The remaining H atoms were positioned geometrically, with C—H distances of 0.93 Å, O—H distance of 0.82 Å, and refined using a riding model, with *U*_iso_(H) = 1.2*U*_eq_(C) or 1.5U_eq_(O).

### Crystal data

**Table:**

C_14_H_10_BrClN_2_O_2_	*F*(000) = 704
*M**_r_* = 353.60	*D*_x_ = 1.691 Mg m^−^^3^
Monoclinic, *P*2_1_/*n*	Mo *K*α radiation, λ = 0.71073 Å
Hall symbol: -P 2yn	Cell parameters from 1461 reflections
*a* = 5.893 (2) Å	θ = 2.5–24.5°
*b* = 31.708 (11) Å	µ = 3.15 mm^−^^1^
*c* = 7.437 (3) Å	*T* = 298 K
β = 92.017 (8)°	Block, colourless
*V* = 1388.8 (9) Å^3^	0.17 × 0.15 × 0.15 mm
*Z* = 4	

### Data collection

**Table:**

Bruker SMART 1000 CCD diffractometer	2661 independent reflections
Radiation source: fine-focus sealed tube	1555 reflections with *I* > 2σ(*I*)
graphite	*R*_int_ = 0.043
ω scans	θ_max_ = 25.9°, θ_min_ = 1.3°
Absorption correction: multi-scan (*SADABS*; Sheldrick, 1996)	*h* = −7→7
*T*_min_ = 0.616, *T*_max_ = 0.649	*k* = −38→36
7670 measured reflections	*l* = −9→5

### Refinement

**Table:**

Refinement on *F*^2^	Primary atom site location: structure-invariant direct methods
Least-squares matrix: full	Secondary atom site location: difference Fourier map
*R*[*F*^2^ > 2σ(*F*^2^)] = 0.041	Hydrogen site location: inferred from neighbouring sites
*wR*(*F*^2^) = 0.119	H atoms treated by a mixture of independent and constrained refinement
*S* = 1.02	*w* = 1/[σ^2^(*F*_o_^2^) + (0.0584*P*)^2^] where *P* = (*F*_o_^2^ + 2*F*_c_^2^)/3
2661 reflections	(Δ/σ)_max_ < 0.001
185 parameters	Δρ_max_ = 0.58 e Å^−^^3^
1 restraint	Δρ_min_ = −0.51 e Å^−^^3^

### Special details

**Table:**

Geometry. All esds (except the esd in the dihedral angle between two l.s. planes) are estimated using the full covariance matrix. The cell esds are taken into account individually in the estimation of esds in distances, angles and torsion angles; correlations between esds in cell parameters are only used when they are defined by crystal symmetry. An approximate (isotropic) treatment of cell esds is used for estimating esds involving l.s. planes.
Refinement. Refinement of F^2^ against ALL reflections. The weighted R-factor wR and goodness of fit S are based on F^2^, conventional R-factors R are based on F, with F set to zero for negative F^2^. The threshold expression of F^2^ > 2sigma(F^2^) is used only for calculating R-factors(gt) etc. and is not relevant to the choice of reflections for refinement. R-factors based on F^2^ are statistically about twice as large as those based on F, and R- factors based on ALL data will be even larger.

### Fractional atomic coordinates and isotropic or equivalent isotropic displacement parameters (Å^2^)

**Table:**

	*x*	*y*	*z*	*U*_iso_*/*U*_eq_	
Br1	0.22343 (9)	0.007519 (14)	0.69748 (8)	0.0934 (3)	
Cl1	−0.31634 (18)	0.42871 (3)	0.73651 (14)	0.0726 (4)	
N1	0.3457 (5)	0.20659 (9)	0.8247 (3)	0.0456 (7)	
N2	0.2126 (5)	0.24185 (9)	0.7948 (4)	0.0508 (8)	
O1	0.7208 (4)	0.16526 (8)	0.9180 (4)	0.0621 (7)	
H1	0.6422	0.1863	0.8999	0.093*	
O2	0.4098 (4)	0.27888 (7)	1.0046 (3)	0.0604 (7)	
C1	0.3853 (6)	0.13282 (10)	0.7884 (4)	0.0418 (8)	
C2	0.6036 (6)	0.13063 (12)	0.8651 (4)	0.0479 (9)	
C3	0.7070 (6)	0.09163 (14)	0.8872 (5)	0.0617 (11)	
H3	0.8539	0.0902	0.9369	0.074*	
C4	0.5988 (7)	0.05541 (13)	0.8381 (5)	0.0665 (11)	
H4	0.6702	0.0295	0.8553	0.080*	
C5	0.3815 (7)	0.05740 (11)	0.7622 (5)	0.0561 (10)	
C6	0.2773 (6)	0.09556 (11)	0.7367 (4)	0.0485 (9)	
H6	0.1320	0.0966	0.6840	0.058*	
C7	0.2645 (6)	0.17218 (11)	0.7635 (4)	0.0446 (9)	
H7	0.1247	0.1723	0.7015	0.054*	
C8	0.2553 (6)	0.27691 (10)	0.8913 (5)	0.0430 (8)	
C9	0.1038 (5)	0.31345 (10)	0.8524 (4)	0.0385 (8)	
C10	−0.1114 (6)	0.30975 (11)	0.7758 (4)	0.0470 (9)	
H10	−0.1700	0.2832	0.7491	0.056*	
C11	−0.2404 (6)	0.34505 (12)	0.7387 (5)	0.0506 (9)	
H11	−0.3850	0.3425	0.6856	0.061*	
C12	−0.1537 (6)	0.38413 (11)	0.7806 (4)	0.0471 (9)	
C13	0.0603 (6)	0.38863 (11)	0.8575 (4)	0.0511 (9)	
H13	0.1184	0.4153	0.8838	0.061*	
C14	0.1861 (6)	0.35333 (11)	0.8947 (4)	0.0465 (9)	
H14	0.3297	0.3561	0.9495	0.056*	
H2	0.107 (5)	0.2405 (12)	0.705 (4)	0.080*	

### Atomic displacement parameters (Å^2^)

**Table:**

	*U*^11^	*U*^22^	*U*^33^	*U*^12^	*U*^13^	*U*^23^
Br1	0.1054 (5)	0.0446 (3)	0.1297 (5)	−0.0038 (2)	−0.0018 (3)	0.0039 (3)
Cl1	0.0765 (8)	0.0562 (7)	0.0844 (8)	0.0210 (5)	−0.0079 (6)	−0.0056 (5)
N1	0.0462 (18)	0.0458 (18)	0.0441 (17)	0.0073 (14)	−0.0094 (13)	−0.0009 (13)
N2	0.055 (2)	0.0462 (19)	0.050 (2)	0.0044 (15)	−0.0177 (14)	−0.0068 (15)
O1	0.0459 (15)	0.0723 (18)	0.0670 (18)	0.0040 (13)	−0.0124 (13)	−0.0054 (15)
O2	0.0676 (17)	0.0470 (15)	0.0639 (17)	−0.0021 (13)	−0.0335 (14)	0.0001 (12)
C1	0.043 (2)	0.046 (2)	0.037 (2)	0.0041 (16)	0.0006 (15)	0.0014 (15)
C2	0.046 (2)	0.057 (2)	0.040 (2)	0.0024 (18)	0.0003 (17)	−0.0009 (17)
C3	0.044 (2)	0.079 (3)	0.062 (3)	0.019 (2)	−0.0053 (18)	0.008 (2)
C4	0.068 (3)	0.060 (3)	0.071 (3)	0.024 (2)	0.003 (2)	0.008 (2)
C5	0.065 (3)	0.047 (2)	0.057 (2)	0.0047 (19)	0.006 (2)	0.0083 (17)
C6	0.048 (2)	0.045 (2)	0.052 (2)	0.0037 (16)	−0.0027 (17)	0.0042 (16)
C7	0.043 (2)	0.048 (2)	0.042 (2)	0.0044 (17)	−0.0053 (16)	0.0019 (16)
C8	0.041 (2)	0.043 (2)	0.045 (2)	−0.0061 (15)	−0.0073 (17)	0.0019 (16)
C9	0.042 (2)	0.0411 (19)	0.0325 (18)	−0.0023 (15)	−0.0044 (15)	−0.0008 (15)
C10	0.051 (2)	0.042 (2)	0.048 (2)	−0.0024 (16)	−0.0004 (17)	−0.0065 (16)
C11	0.048 (2)	0.053 (3)	0.051 (2)	0.0031 (18)	−0.0027 (17)	−0.0043 (17)
C12	0.056 (2)	0.045 (2)	0.041 (2)	0.0075 (17)	0.0085 (17)	−0.0042 (16)
C13	0.060 (3)	0.041 (2)	0.052 (2)	−0.0033 (18)	0.0023 (18)	−0.0062 (17)
C14	0.045 (2)	0.050 (2)	0.045 (2)	−0.0046 (17)	−0.0068 (16)	−0.0041 (16)

### Geometric parameters (Å, °)

**Table:**

Br1—C5	1.889 (4)	C4—H4	0.9300
Cl1—C12	1.733 (4)	C5—C6	1.367 (5)
N1—C7	1.269 (4)	C6—H6	0.9300
N1—N2	1.379 (4)	C7—H7	0.9300
N2—C8	1.342 (4)	C8—C9	1.485 (4)
N2—H2	0.898 (10)	C9—C10	1.377 (4)
O1—C2	1.349 (4)	C9—C14	1.387 (4)
O1—H1	0.8200	C10—C11	1.376 (5)
O2—C8	1.220 (4)	C10—H10	0.9300
C1—C6	1.390 (5)	C11—C12	1.372 (5)
C1—C2	1.391 (5)	C11—H11	0.9300
C1—C7	1.445 (5)	C12—C13	1.373 (5)
C2—C3	1.386 (5)	C13—C14	1.366 (5)
C3—C4	1.358 (5)	C13—H13	0.9300
C3—H3	0.9300	C14—H14	0.9300
C4—C5	1.383 (5)		
			
C7—N1—N2	115.7 (3)	N1—C7—H7	119.4
C8—N2—N1	119.4 (3)	C1—C7—H7	119.4
C8—N2—H2	123 (3)	O2—C8—N2	122.2 (3)
N1—N2—H2	117 (3)	O2—C8—C9	121.6 (3)
C2—O1—H1	109.5	N2—C8—C9	116.2 (3)
C6—C1—C2	118.6 (3)	C10—C9—C14	118.8 (3)
C6—C1—C7	118.7 (3)	C10—C9—C8	123.6 (3)
C2—C1—C7	122.7 (3)	C14—C9—C8	117.7 (3)
O1—C2—C3	118.3 (3)	C11—C10—C9	120.6 (3)
O1—C2—C1	122.4 (3)	C11—C10—H10	119.7
C3—C2—C1	119.3 (3)	C9—C10—H10	119.7
C4—C3—C2	121.5 (4)	C12—C11—C10	119.3 (3)
C4—C3—H3	119.2	C12—C11—H11	120.3
C2—C3—H3	119.2	C10—C11—H11	120.3
C3—C4—C5	119.4 (4)	C11—C12—C13	121.2 (3)
C3—C4—H4	120.3	C11—C12—Cl1	119.6 (3)
C5—C4—H4	120.3	C13—C12—Cl1	119.2 (3)
C6—C5—C4	120.1 (4)	C14—C13—C12	118.9 (3)
C6—C5—Br1	119.4 (3)	C14—C13—H13	120.5
C4—C5—Br1	120.5 (3)	C12—C13—H13	120.5
C5—C6—C1	121.0 (3)	C13—C14—C9	121.2 (3)
C5—C6—H6	119.5	C13—C14—H14	119.4
C1—C6—H6	119.5	C9—C14—H14	119.4
N1—C7—C1	121.3 (3)		

### Hydrogen-bond geometry (Å, °)

**Table:**

*D*—H···*A*	*D*—H	H···*A*	*D*···*A*	*D*—H···*A*
O1—H1···N1	0.82	1.93	2.642 (4)	145
N2—H2···O2^i^	0.90 (1)	1.96 (2)	2.829 (3)	163 (4)

Symmetry codes: (i) *x*−1/2, −*y*+1/2, *z*−1/2.
